# Add-on ketogenic diet versus antiseizure medications alone in children with developmental and epileptic encephalopathies: a prospective comparative cohort study

**DOI:** 10.3389/fneur.2025.1677046

**Published:** 2025-10-07

**Authors:** Wandong Hu, Lili Li, Fen Zhao, Song Su, Hongwei Zhang, Xiaoying Li

**Affiliations:** ^1^Department of Neurology, Children’s Hospital Affiliated to Shandong University, Jinan Children’s Hospital, Jinan, China; ^2^Department of Neonatology, Children’s Hospital Affiliated to Shandong University, Jinan Children’s Hospital, Jinan, China

**Keywords:** ketogenic diet, antiseizure medications, developmental and epileptic encephalopathies, effectiveness, safety

## Abstract

**Objective:**

To compare the effectiveness and safety of ketogenic diet (KD) versus antiseizure medications (ASMs) adjustment in developmental and epileptic encephalopathy (DEE).

**Methods:**

In this prospective, single-center cohort study, 268 participants were allocated to either the KD group (*n* = 128) or the control group (*n* = 140; ASMs adjustment alone). Outcomes were assessed at 3 and 6 months. The primary outcome was the ≥50% seizure response rate at 6 months. Secondary outcomes included seizure-free rates, electroencephalography (EEG) improvements, and developmental progress. Other outcomes included adverse events, retention rate, and predictors of KD response.

**Results:**

At 6-month evaluation, KD group demonstrated significantly better seizure response than in controls (50.78%vs.29.29%RR = 1.73, 95% CI 1.27–2.36, *p* < 0.001). The KD group superior in secondary outcomes, including higher seizure-free rates, greater EEG improvement, and better developmental progress. The adverse actions were slight and acceptable. Survival analysis revealed a higher retention rate in KD group than that in control group at any point (HR = 0.68, 95%CI 0.50–0.92, *p* < 0.05). No significant predictor of KD effectiveness could be found.

**Interpretation:**

KD demonstrated superior effectiveness and safety over ASMs adjustments in children with DEE, achieving not only reduced seizure frequency and EEG abnormalities but also improved developmental outcome. These findings supported early consideration of KD in DEE management.

## Introduction

Developmental and epileptic encephalopathy (DEE) represents a spectrum of severe neurodevelopmental disorders characterized by frequent seizures and developmental delay, and caused by underlying etiologies (1). First formally proposed by the International League Against Epilepsy (ILAE) in 2017 (2) and introduced with an operational definition in 2025 (1), DEEs typically manifest in early childhood with drug-resistant epilepsy and have the high morbidity and mortality rates (3). With the continual introduction of new anti-seizure medications (ASMs), most children with DEE still suffer from uncontrolled seizures. These conditions could cause poor quality of life of children and heavy economic burden to their family, which has become a serious social problem. Therefore, seeking a favorable efficacy treatment was extremely urgent for children with DEE.

The ketogenic diet (KD), first introduced as a therapeutic intervention for drug-resistant epilepsy in the 1920s (4), remains a cornerstone of non-pharmacological treatment for drug-resistant epilepsy. This rigorously formulated dietary regimen, characterized by a high-fat, low-carbohydrate, and adequate protein composition, has gained Level A evidence status from the ILAE for its efficacy in drug-resistant epilepsy (5). The emerging evidence has been accepted that KD impact on multiple epileptogenic processes and targets, such as the changes in neurotransmitter systems and channel regulation, enhancement of cellular bioenergetics and mitochondrial function (6).

In recent years, emerging evidence has established the therapeutic potential of KD for specific DEEs, such as Dravet syndrome (7) and Infantile epileptic spasm syndrome (IESS) (8). However, these current literatures focus on the retrospective study designs or lack of contrast with receiving ASMs adjustment alone. Therefore, we designed this prospective cohort study to compare the effectiveness and safety of KD versus ASMs adjustment alone in children with DEE, in order to summarize clinical experience of KD treatment for DEE and better guide pediatric practice.

## Methods

### Participants

All children with DEE who received KD or ASMs adjustment between were recruited January 2021 and January 2024 at the Children’s Hospital Affiliated to Shandong University. The study was approved by the Ethics committee of Children’s Hospital Affiliated to Shandong University (No. SDFE-IRB/P-2022047). Written informed consent was obtained from all legal guardians prior to study participation.

The inclusion criteria required that (1) age ≤18 years old; (2) definitive diagnosis of DEE according to the latest operational definition in 2025 (1), the specific DEE classification according to ILAE nosology and definition of epilepsy syndromes in 2022 (9); (3) suffering from uncontrolled seizures with two or more appropriate ASMs; (4) never exposing KD treatment previously; (5) minimum follow-up duration of 6 months. And we also exclude those with the following situations: (1) those inapplicable to KD who inborn with metabolic diseases, progressive neurological disorders, and severe gastroesophageal reflux disease; (2) Incomplete baseline or follow-up data (>20% missing variables); (3) those with poor cooperation of patients or their families.

All enrolled patients were non-randomly assigned to either the KD group or control group based on comprehensive clinical assessment by specialized doctors and family consent. The KD group initiated a classical 4:1 ratio ketogenic diet under close medical supervision, maintaining baseline ASMs without dose escalation, while implementing strict ketone monitoring and micronutrient supplementation. In contrast, the control group continued conventional ASM management with medication adjustments while maintaining their regular non-ketogenic diet. Both groups were followed prospectively for treatment outcomes, with the KD group undergoing gradual dietary induction over 5–7 days and both groups receiving regular clinical monitoring.

Based on prior data demonstrating a 54.1% seizure reduction rate with KD versus 31.6% with ASMs adjustment in Chinese pediatric patients with IESS over 16 weeks (10), we performed an *a priori* power analysis using PASS software. Assuming *α* = 0.05 (two-tailed) and *β* = 0.20 (80% power) for detecting this effect size, the calculated minimum required sample size was 184 participants (92 per group). Therefore, our final enrollment of 268 samples (KD group: *n* = 128; control group: *n* = 140) exceeded this requirement.

### Study type

This was a single-center, prospective cohort study. Before starting this study, we designed the tables to collected data, including sex, age, family history, birth history, age of seizure onset, seizure frequency, seizure type, duration of disease, discharge site in EEG, structural abnormality in MRI, epileptic syndrome, the time of starting KD, and ASMs adjustment. The information about seizure outcomes was extracted from interviews with parents and patient diaries reviewed during following up. The follow-up time points were 3 months and 6 months. The duration of follow-up was 6 months after KD initiation or until the diet was discontinued, whichever occurred first.

Primary outcome data were obtained through structured parental interviews and validated seizure diaries, with comprehensive evaluations conducted at standardized 3-month and 6-month follow-up intervals. The observation period continued for 6 months post-treatment initiation or until KD discontinuation, ensuring consistent endpoint assessment across all participants.

### Definitions

The primary outcome of the study was seizure response rate at 6 months follow-up. Seizure response rate was defined as the proportion of patients whose seizure frequency achieve greater than 50% seizure reduction compared to baseline. Secondary outcomes included seizure response rate at 3 months follow-up; seizure-free rates, electroencephalography (EEG) improvements, developmental progress, and adverse events at the two follow-up endpoints. Seizure-free rate was defined as the proportion of patients without seizure compared to baseline. EEG improvement rate was defined as the proportion of patients whose epileptic discharges achieve greater than 50% discharge reduction in the follow-up EEG compared to baseline. Development Improvement was measured by development scales according to different age groups, such as GESELL scales (0–6 years old). During each routine follow-up, the same development scale as in the baseline evaluation was selected for assessment. And we try to select the same specialist as much as possible to minimize bias. If the scores of development scales in the follow-up are better than in the baseline, we record it as development improvement. Adverse events were evaluated based on parental observations and descriptions.

### Diet administration

Patients were evaluated by clinicians and nutritionists 10 days or more before receiving KD (baseline). The pre-diet assessment consisted of nutritional evaluation including baseline weight and height, blood biochemical items including serum lipids and albumin, and urologic ultrasound to rule out contraindications to KD. The KD was started without prior fasting as add-on therapy to daily ASMs. We adopt the classic KD regimen, with a ketogenic ratio of 4:1 (3:1 in infants <1 year). The necessary intake of calories was established by a nutritionist based on the nutritional status and level of physical activity of the participants and complied with the Chinese recommended intake for age and weight. Adverse effects were monitored, and the KD regimen was adjusted accordingly by Nutritionist to maintain the child’s blood ketones at 3–5 mmol/L and blood glucose at 4–5 mmol/L. The final personalized diet was developed with the goal of controlling seizures and the best possible quality of life. The KD maintenance period was at least 1 month to assess effectiveness.

All participants were followed up through outpatient clinic visits and telephone calls. Parents record daily diaries, including the seizure frequency, weight, height, urine ketone, blood glucose, and adverse events. Follow-up evaluations were scheduled at the 3rd and 6th months.

### Statistical analysis

All the statistical analyses in this study were performed using SPSS Statistics version 26. Results are expressed as number and percentages for categorical variables, as the mean ± standard deviation (SD), or median with interquartile range (IQR) for continuous variables. Differences between groups were analyzed using non-parametric tests (Mann–Whitney U test for continuous variables) or *χ*^2^ Fisher’s exact test for categorical variables. Survival analysis was conducted by setting the follow-up time as the time variable, the retention rate as the outcome variable, KD or ASMs adjustments as the independent variable. HR represents the hazard ratio, and 95%CI represents the 95% confidence interval. Univariate analysis was performed to explore the predictors of KD effectiveness at 6-month following up. All statistical results were considered statistically significant with *p* < 0.05.

## Results

### Study population

From January 2021 to January 2024, a total of 296 children were started on the KD at our institution. Of those patients, 18 patients from the KD group who persist in KD treatment for less than 6 months and 10 patients from the control group who were lost to follow-up were excluded. Finally, the remaining 268 patients, of whose 128 in the KD group and 140 in the control group, were included in our study. The baseline demographic data and clinical details of our cohort are listed in Table 1. No statistically significant difference between the two groups was found (*p* > 0.05). In addition, among 61 patients with obtaining genetic information in KD group, 25 patients have specific pathogenic gene variants, including SCN1A in ten cases, MECP2 in three cases, deletion in three cases, SCN2A in two cases, DEPDC5 in two cases, KCNT1 in one case, CDKL5 in one case, STXBP1 in one case, DYNC1H1 in one case, KCNQ1 in one case. In control group, among 52 patients with obtaining genetic information, 11 patients have specific pathogenic gene variants, including SCN1A in two cases, NF1 in two cases, SCN2A in one case, GABRB3 in one case, NALCN in one case DMN1 in one case, GATA5 in one case, TSC2 in one case, and deletion in one case. Given that the above genetic data was scattered and could not be subjected to statistical analysis, we did not present it in the Table 1.

**Table 1:** Baseline demographics and clinical traits of the comparison between two groups.

Baseline patient demographics and clinical traits	KD group (*N* = 128)	Control group (*N* = 140)	*p* value
Sex, *n* (%)			
Male	80 (62.50%)	83 (59.29%)	0.59
Female	48 (37.50%)	57 (40.71%)	
Age at seizure onset (months)	9.35 (4.40, 24.10)	6.50 (5.00, 16.07)	0.27
Duration of disease (months)	8.15 (3.35, 23.60)	9.45 (5.10, 16.35)	0.31
Age at initiation of KD (months)	22.08 (11.58, 54.73)	–	–
Baseline seizure frequency (times/month)	4.00 (3.00, 8.00)	4.00 (2.00, 7.00)	0.30
Seizure type			
Focal	34 (26.56%)	25 (17.86%)	0.20
Generalized	70 (54.69%)	82 (58.57%)	
Both	24 (18.75%)	33 (23.57%)	
Epilepsy syndromes			
IESS	62 (48.44%)	66 (47.14%)	0.96
DS	15 (11.72%)	18 (12.86%)	
LGS	9 (7.03%)	8 (5.71%)	
NOS	42 (32.81%)	48 (34.29%)	
Discharge in EEG			
Focal	36 (28.12%)	27 (19.29%)	0.23
Generalized	64 (50.00%)	80 (57.14%)	
Both	28 (21.88%)	33 (23.57%)	
Family history^a^	12 (9.38%)	10 (7.14%)	0.51
Number of ASMs at baseline	3.00 (3.00, 3.00)	3.00 (3.00, 4.00)	0.37

Data are *n* (%) or m (IQR).

IESS, Infantile epileptic spasms syndrome; DS, Dravet syndrome; LGS, Lennox–Gastaut syndrome; NOS, not otherwise specified; EEG, electroencephalogram; ASMs, anti-seizure medications.

a Family history referred to the presence of one or more close biological relatives who have been diagnosed with epilepsy or a history of epileptic seizures.

### Treatment effectiveness

**Figure 1:**
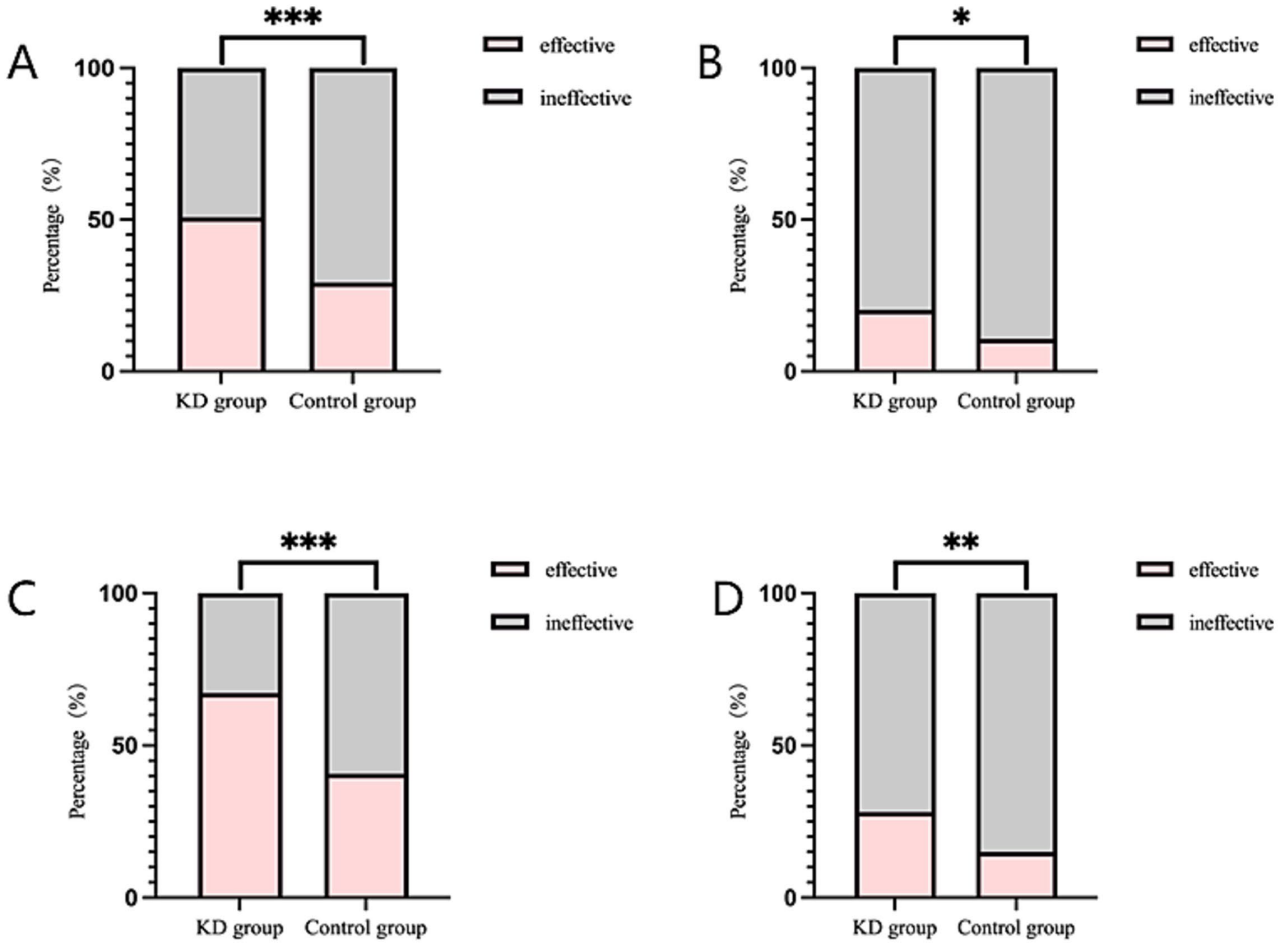
Comparison of seizure response rate and seizure-free rate between the two groups. **(A)** Seizure response rate in 6-month following up. **(B)** Seizure-free rate in 6-month following up. **(C)** Seizure response rate in 3-month following up. **(D)** Seizure-free rate in 3-month following up. *indicates *p* < 0.05; **indicates *p* < 0.01; ***indicates *p* < 0.001.

In primary outcome, the seizure response rate in KD group was higher than that in the control group at 6 months of follow-up (50.78% vs.29.29%), and the difference was statistically significant (RR = 1.73, 95%CI 1.27–2.36, *p* < 0.001) (Figure 1A).

**Figure 2:**
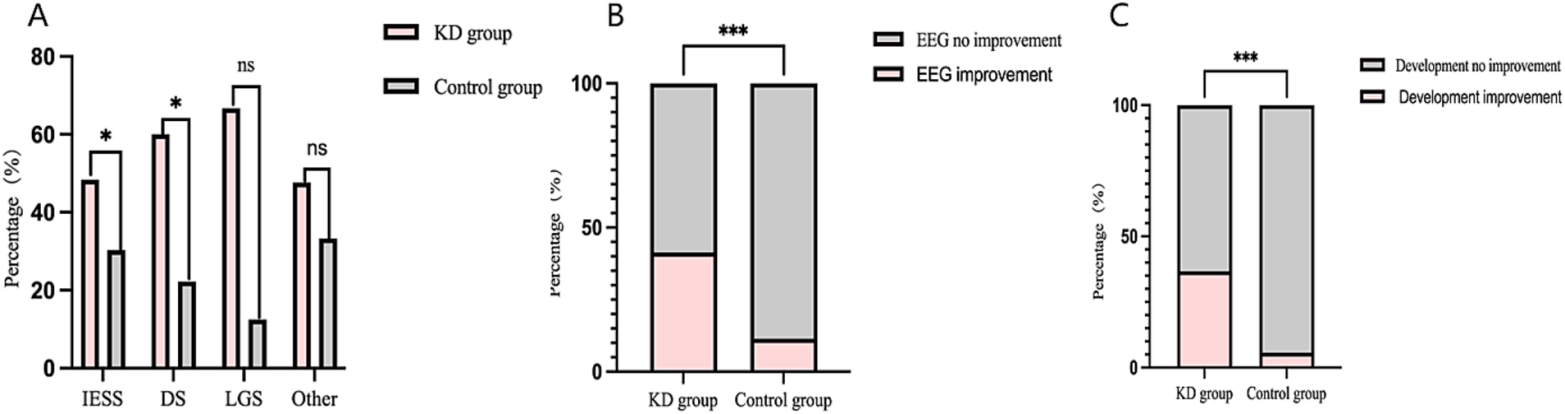
**(A)** Comparison of seizure response rate in different epilepsy syndromes between the two groups in 6-month following up. **(B)** Comparison of EEG improvement rate in between the two groups in 6-months following up. **(C)** Comparison of rate in developmental improvement rate between the two groups in 6-months following up. *indicates *p* < 0.05; **indicates *p* < 0.01; ***indicates *p* < 0.001.

Among secondary outcomes, the seizure-free rate in the KD group was also higher than that in the control group at 6 months of follow-up (20.31%vs.10.71%), and the difference was statistically significant (RR = 1.90, 95%CI 1.05–3.42, *p* < 0.05) (Figure 1B). At 3 months of follow-up, the seizure response rate and seizure-free rate were higher than those in the control group (67.19% vs.40.71, 28.13%vs.15.00%, respectively), and the both differences were statistically significant (RR = 1.65, 95%CI 1.31–2.09, *p* < 0.001; RR = 1.88, 95%CI 1.16–3.04, *p* < 0.01, respectively) (Figures 1C,D). Among epilepsy syndromes (Figure 2A), we found that the seizure response rate of IESS and Doose syndrome (DS) diagnosis in KD group were significantly higher than that in the control group at 6 months of follow-up (48.39%vs. 30.30, 60.00% vs. 22.22%, all *p* < 0.05). However, the difference of seizure response rate in LGS diagnosis between two groups was not statistically significant. In addition, the EEG improvement rate in the KD group was significantly higher than that in the control group at 6 months of follow-up (41.41% vs.11.43%, *p* < 0.001) (Figure 2B). Similarly, the developmental improvement rate in the KD group was significantly higher than that in the control group (36.72% vs. 5.71%, *p* < 0.001) (Figure 2C).

### Adverse reactions of KD

A total of 45 patients (35.16%) in KD group experience adverse reactions. Among them, 41 (32.03%) patients had gastrointestinal reactions, including 20 (15.63%) of vomiting, 12 (9.38%) of diarrhea, and 9 (7.03%) of constipation. But these adverse reactions were slight and tolerable, which could be improved by adjusting the proportion of diet and sample treatment. Only 4 children (3.13%) discontinued KD treatment, of which 3 cases (2.34%) because of hyperuricemia and 1 case (0.78%) because of urinary calculi. All observed adverse events occurred during the first 3 months of KD treatment.

### Retention rate

**Figure 3:**
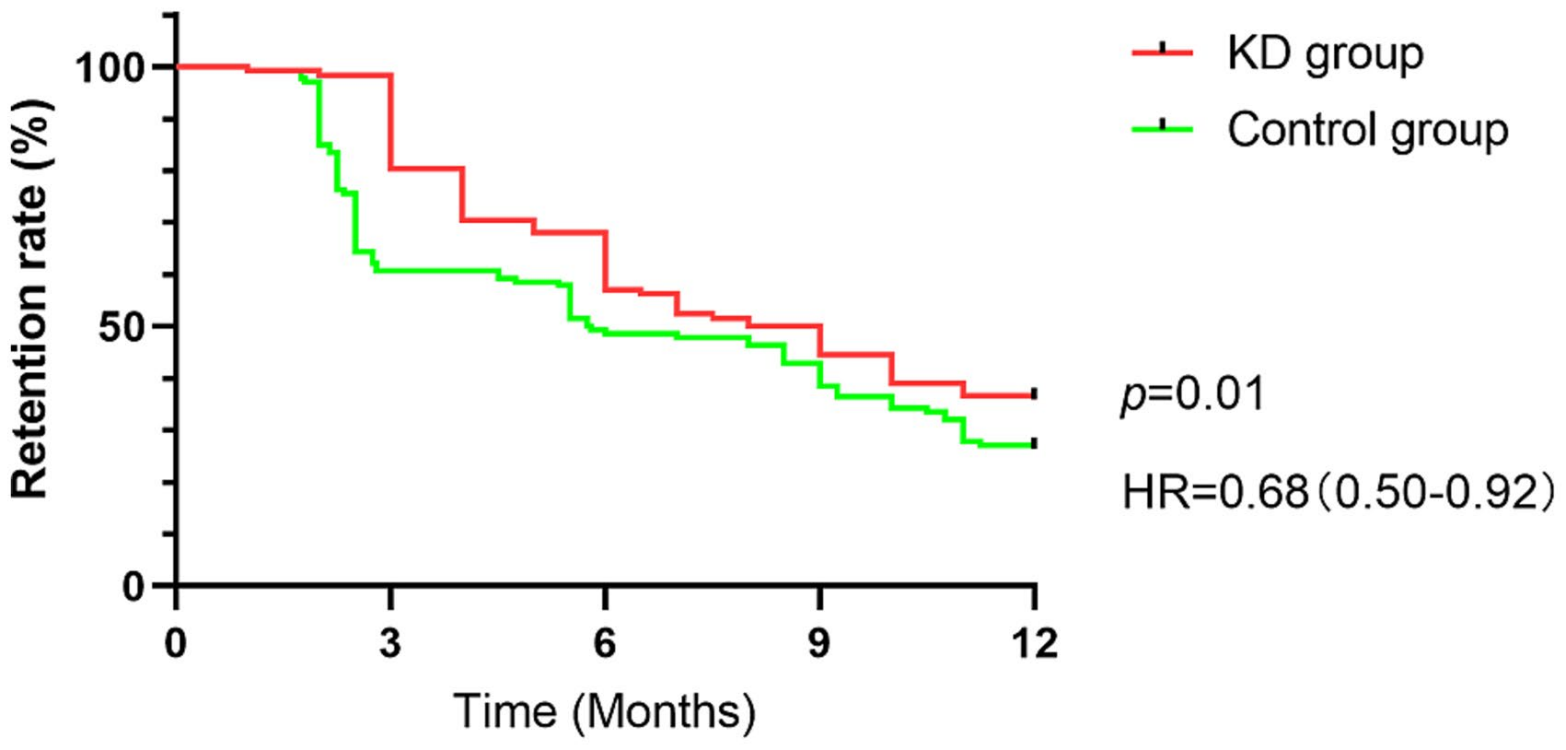
The survival curve of retention rate between the two groups.

The median retention time was 8.50 months in the KD group and 5.78 months in the control group. Survival analysis showed that retention rate in KD group was better than that in control group at any point during one-year following up (HR = 0.68, 95%CI 0.50–0.92), with statistically significant difference (*p* = 0.01) (Figure 3).

### Factors influencing of KD effectiveness

Univariate analysis was performed by setting seizure response of KD treatment at 6 months of follow-up as the dependent variable, and gender, age at seizure onset, duration of disease, seizure type, epilepsy syndrome, hypsarrhythmia on EEG, EEG discharge type, etiologies, seizure frequency, family history, numbers of ASMs at baseline, age at onset of KD, and ketone body level as independent variables. No statistical factor was found to influence effectiveness of KD treatment (*p* > 0.05).

## Discussion

Our study demonstrates the superior effectiveness of KD treatment compared to ASMs adjustment in children with DEE, as evidenced by significant reductions in seizure frequency, improvement in EEG abnormalities, and measurable developmental gains. Furthermore, the mild adverse reactions and the high retention rate detected in our study suggested that KD treatment with favorable tolerability and acceptable safety. Therefore, early initiation of KD may be considered for those children with DEE unresponsive to multiple ASMs. To our knowledge, the design of the prospective cohort study and setting those receiving ASMs adjustment as a control in our study were the novel perspectives of clinical evaluation of KD treatment in Chinese children with DEE.

Published data about KD treatment for DEE are limited to one certain epilepsy syndrome but generally encouraging. In overall, we found the 6-month effective rate of KD treatment for DEE was up to 50.78%, which was consistent with previous systematic reviews (11) showing that half of the patients have a better than 50% improvement in seizures with KD. In 3-months follow-up, our results showed the frequency of seizures in 67.19% of patients could be reduced by more than 50%, and about 28.13% had seizure control. A previous study about newborns and infants under 3 months of age with DEE indicated that the effective rate of KD treatment was 72.2% and seizure-free rate was 21% at 3-month follow-up (12). What is more, we also found the effective rate has gradually decline over time, as both the effective rate and the seizure-free rate showed a downward trend in the three follow-up periods. Correspondingly, the retention rate also showed a downward trend, but of which in KD group was superior to that in the control group at each time point. This phenomenon reminded us that KD treatment should be add to children with DEE as early as possible.

In terms of certain epilepsy syndrome, our results showed 48.39% of children with IESS were treated effectively by KD, which similar to previous studies (8). A retrospective study about 119 children with IESS from China that 47.9% exhibited effective seizure reducing by 16-week KD treatment (13). In Dravet syndrome, about 60.00% had effective KD treatment, which also was within the effective scope of 58–70% indicated from the previous studies (7, 14). Even, a prior retrospective study found efficacy and tolerability of KD treatment were superior than various ASMs (15), suggesting that KD should be considered as an early treatment option in DS, which was similar to our results. However, our results showed a higher efficacy of 66.67% in children with LGS after 6-month KD treatment, than the 47% in the previous studies (16, 17). And we found no significant difference of efficacy between KD treatment and ASMs adjustment in LGS. The possible reasons of this discrepancy we suppose was that our small sample size of LGS, and increasing the sample size may yield more accurate results.

As a genetically heterogeneous disease, DEE in our study present a variety of pathogenic gene variants. Among them, the most common were related to ion channel, such as SCN1A, KCNQ2, STXBP1, and CDKL5, which was consistent with the previous study (18). And our results showed KD treatment presented a good effectiveness for DEE with these genes. The mechanism of KD treatment for these DEE involved multiple pathways of regulating synaptic level ion channels and transporter proteins to reduce neuronal excitability (19, 20). For example, the production of ketone bodies could decrease activity of ATP-sensitive potassium channels to reduce neuronal cell excitability (21) or modulate vesicle fusion to affect inter-neuronal neurotransmitter release (22).

In addition, we also found KD could reduce abnormal discharges in EEG and improved the development of children with DEE. In our study, approximately 41.41% of patients with DEE receiving KD achieved a reduction of over 50% in the frequency of abnormal discharges in the 6-month follow-up, which was consistent with previous studies (23, 24). Moreover, in the early stage of KD treatment, such as 3 months of following up, we observed a reduction of interictal discharges in EEG. A prior study has also found that the interictal discharge index of 24-h EEG has decreased significantly at 6 weeks of KD treatment (24). Furthermore, KD treatment may also help improve the sleep cycle and circadian rhythm of children with DEE. In an animal experiment about KCNA1-deficient mice with epilepsy, KD treatment reduced seizure frequency, as well improved the sleep cycle and circadian rhythm of mice (25). But these situations whether exist in children with DEE still require further researches. Meanwhile, our results showed 36.72% of patients gained developmental and cognitive improvement in the 6-month follow-up. In fact, the association between KD treatment and developmental improvement has been discovered in previous studies (26), and more significant improvement might be obtained with prolonged KD treatment (27). Therefore, as a neurodevelopmental disorder, DEE urgently required the KD treatment to achieve both reducing seizure frequency and improving development.

Our results also showed the number of patients who adhere to KD treatment decreases with the extension of follow-up time, which was consistent with previous study (28). This attrition likely reflects multiple factors including dietary intolerance, adverse reactions, and decline in effectiveness over extended periods. Specifically, the retention rate in KD group was 68% in the 6-months following up, which was consistent with previously published reports from Asian countries (7, 14) and even Western countries, although the customary diet for our country’s population contains substantially less fat than does the traditional Western diets. Just as the consensus stated (23), flexibility in the initiation of KD is well-supported based on clinical practices, and many professors no longer prescribes fasting at KDT onset. This further reduces the adverse action in the initiation of KD and enhances the compliance of patients. Importantly, our survival analysis showed consistently superior retention rates in the KD group versus ASMs adjustments alone at all evaluation timepoints. These findings robustly confirmed the clinical preference and sustained tolerability of KD treatment in for children with DEE.

Finally, the whole adverse reactions of KD treatment were slight and acceptable, gastrointestinal symptom was usually most common and the earliest discovered short-term adverse reaction. In the current study, approximately one-third of children developed gastrointestinal symptoms in the early stage of add-on KD treatment, such as vomiting, diarrhea, and constipation. However, these symptoms were effectively improved within 3 months through appropriate dietary modifications. Regarding the long-term observation, several metabolic events, including hyperlipidemia, hyperuricemia, kidney stones, and growth and development disorders, also occurred occasionally (23). Notably, we did not observe sever hyperlipidemia. Slight hyperlipidemia generally did not require special intervention. Treatment discontinuation occurred in four cases, that is three due to hyperuricemia and one due to urinary stones. In patients with maintaining KD treatment, high acid urine level in conjunction with low fluid intake, could increase the risks of ureteral stone formation (29). Previous study showed the incidence of kidney stones in patients treated with KD was 2.2–6.7% (30). Importantly, most cases of kidney stones can be managed conservatively with potassium citrate supplementation and increasing fluid intake, with only rare instances requiring surgical intervention. Additionally, due to the restriction of protein and total calories, a strictly proportioned KD may affect the growth and development of children (31), which likely related to osteopenia due to low intake of vitamin D and calcium (32). Therefore, vitamin D and calcium supplementation is important for pediatric patients during KD treatment.

Our study has several limitations. First, the treatment allocation was based on clinical decision-making rather than randomization, which may exist some selection bias and confounding factors. Nevertheless, no difference was found when comparing the baseline data of two groups. Second, the sample size, though substantial for a single-center study of a rare condition, limits the statistical power for subgroup analyses and for detecting less common adverse effects. Hence, we did not make a specific classification of focal seizure and generalized seizure. Larger samples or multicenter researches are need in the future. Third, the EEG and development examination at the baseline and each follow-up may introduce the potential for assessment bias, particularly for subjective outcome measures. Finally, the follow-up period may be insufficient to evaluate the long-term sustainability and adverse reactions of KD treatment. Future long-term follow-up with >2 years trials are warranted to validated it.

## Conclusion

In conclusion, our findings suggested that KD treatment was superior to ASMs adjustment for children with DEE, reflecting not only reducing seizure frequency and EEG abnormalities but also improving developmental outcomes. The favorable safety profile, evidenced by mild adverse actions and high retention rates, further supported KD’s clinical utility. Therefore, early initiation of KD treatment might be a good therapeutic strategy for those children with DEE unresponsive to multiple ASMs.

## Data Availability

The original contributions presented in the study are included in the article/supplementary material, further inquiries can be directed to the corresponding authors.
